# On the Reappearance of Contagious Diseases

**Published:** 1803-08-01

**Authors:** W. Marson

**Affiliations:** Member of the College of Surgeons


### *On the* Reappearance *of* Contagious Diseases.

I thank you for your flattering notice of my paper, but I believe you misunderstood me; when I spoke of having more observations, I did not mean to say they were all on the subject of the sick chamber, but those arising during some years of practice in Surgery, Midwifery, and the Practice of Physic.

There is a proposal for the extermination of the Small-pox by universal vaccination; but to me there seems something more than Vaccination, and the plan already mentioned, necessary to extirpate that disease.

Some years ago, in looking over the Philosophical Transactions, I read, that during a funeral at a country village, there was a most abominable stench issued from the grave, which was found to proceed from the bursting of a coffin, and which, to the best of my recollection, the hoary sexton declared to have been deposited nearly twenty years; many of the people went home sick, and in a short time the small-pox was very general in the village.

Now, Gentlemen, if the variolous infection may lay dormant in the earth so many years, it certainly would be politic for the grave-digger to make use of some fumigating process at the time of his occupation, to annihilate the small-pox infection, provided there should be any extricated; and unless it is by some means destroyed, it is probable that we may be frequently exposed to the contagion.

I am, &c.

W. MARSON,

Member of the College of Surgeons.

